# Gain weight and sleep desynchronization in workers of a tertiary hospital

**DOI:** 10.1186/1758-5996-7-S1-A124

**Published:** 2015-11-11

**Authors:** Maria Carlota Borba Brum, Camila Morelatto de Souza, Claudia Carolina Schnorr, Fábio Fernandes Dantas Filho, Gustavo Borchardt Bottega, Sheila Stoniosso, Karen Gomes Avila, Otávio Azevedo Bertoletti, Lisandra Almeida Nunes, Ticiana C Rodrigues

**Affiliations:** 1 SBEM, Porto Alegre, Brazil

## Background

The effects of night work or shift work on workers' health are unknown, recent findings have indicated that may affect glucose tolerance, lead to obesity, diabetes and metabolic syndrome (MS). The desynchronization of the circadian cycle has been related to some of these effects, as well as sleep deprivation and exposure to light at night.

## Objectives

To study the association between shift work and chronic diseases and quality of life among health professionals of a university hospital and compare workers day and night shifts in relation to metabolic changes and altered sleep pattern.

## Materials and methods

Cross-sectional study conducted between April 2013 and December 2014. Sociodemographic data were evaluated and for the quality of life we used the WHOQOL BREF. Cronotypes and daily preferences sleep were investigated by Chronotype Questionnaire Munich (MCTQ ). Sleep quality was assessed by questionnaire Pittisburgh Sleep Quality Index. Physical examination was performed and venous blood was collected in fasting for 12 h for laboratory analysis.

## Results

**Figure 1:**
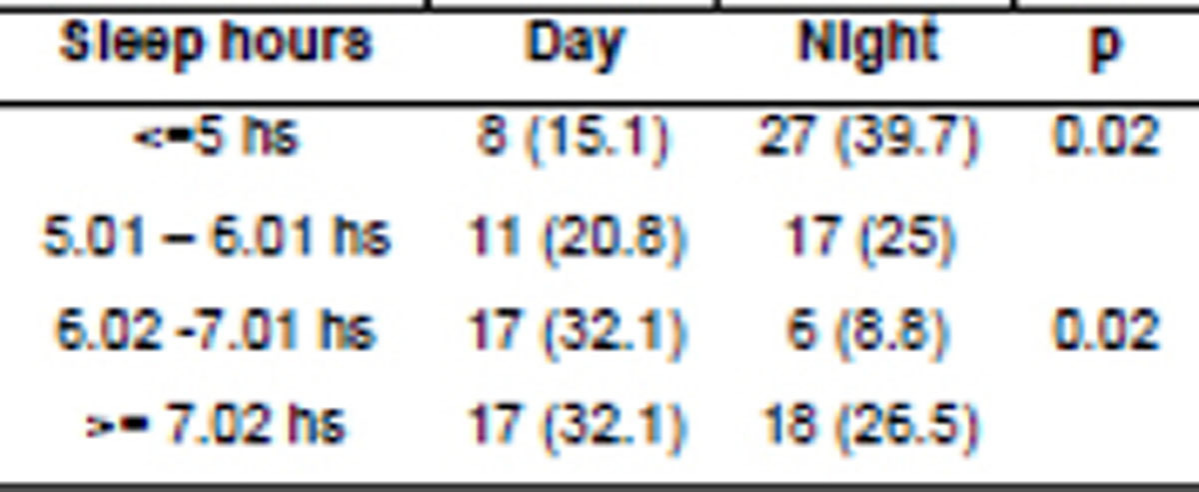
Number of hours of sleep health professionals get in relation to the shift.

**Figure 2:**
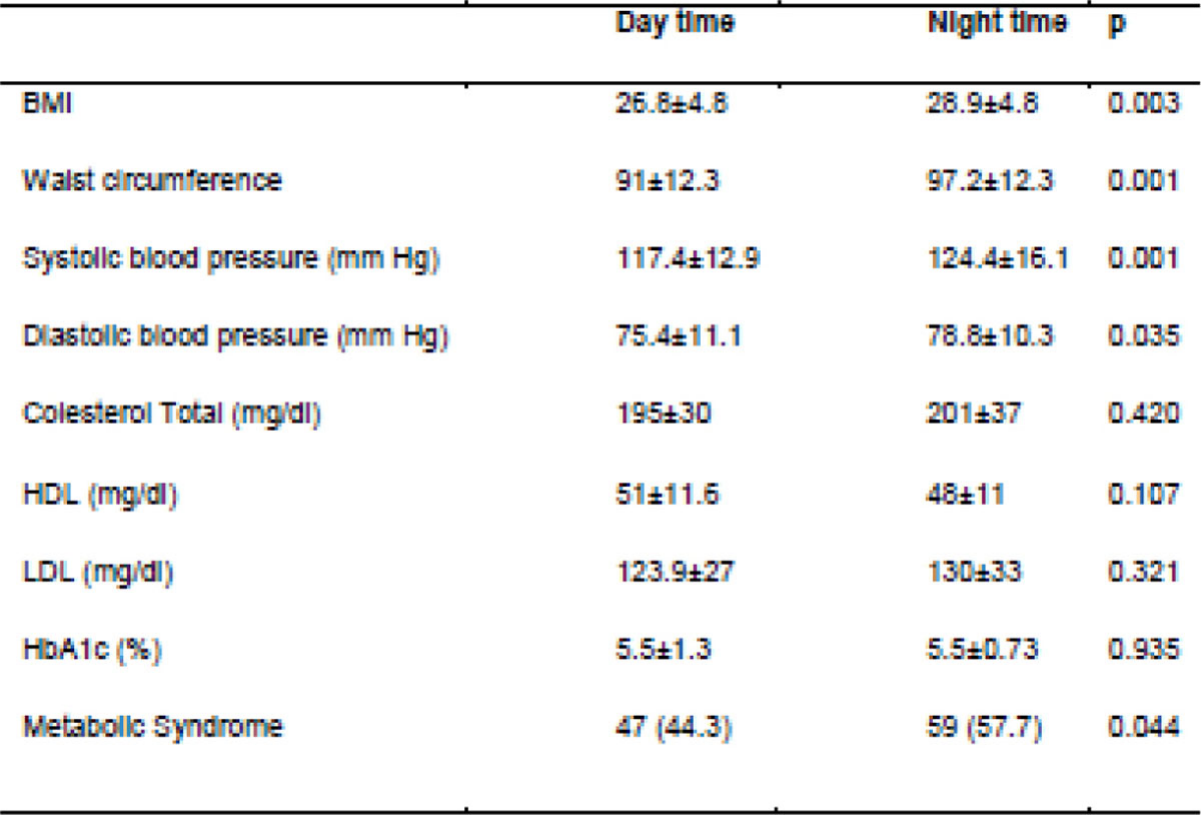
Clinical characteristics of patients in relation to the shift work.

**Figure 3:**
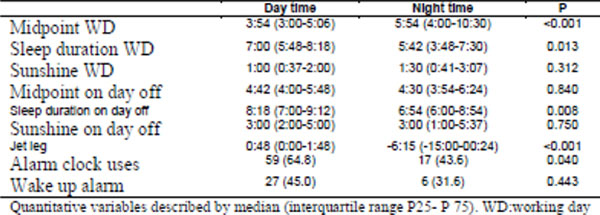
Characteristics for the chronotype and duration of sleep.

129 women and 49 men were included, 108 of the day shift and 80 from night. Night workers had more income, were older, had more time in the institution, less sleep in h, higher BMI, larger waist circumference, higher prevalence of MS and higher levels of blood pressure (Figure 1 and 2) in comparison with daytime workers. There was no difference regarding glucose levels, insulin, HOMA-IR and lipid profile. Figure 3 shows the MCTQ data, observing statistically significant difference between the midpoint on working days, the duration of sleep on days off, on jetleg and use of alarm between daytime and night workers. A negative correlation between the Jet-lag (weekly sleep deficit) and BMI and waist (r: -0.21, p=0.01 and r: -00: 27, p=0.003, respectively) was reported. In regression analysis, adjusted for age and sex, sleeping less than 5 h/24 hs or work at night have been associated with excess body weight (OR: 3.65,95%CI: 1: 02 to 13: 01, p=0.01 and OR: 2.35,95%CI: 1.14-4.84, p=0.02 respectively).

## Conclusions

The reduction of h slept or desynchronization of sleep (night work) can be a possible mechanism in the pathogenesis of obesity and should be better understood.

